# Patterns and drivers of movement for a coastal benthopelagic fish, *Pseudocaranx georgianus*, on Australia’s southeast coast

**DOI:** 10.1038/s41598-018-34922-6

**Published:** 2018-11-13

**Authors:** Ashley M. Fowler, Rowan C. Chick, John Stewart

**Affiliations:** 1 New South Wales Department of Primary Industries, Sydney Institute of Marine Science, Mosman, NSW 2088 Australia; 2 New South Wales Department of Primary Industries, Port Stephens Fisheries Institute, Taylors Beach, NSW 2316 Australia

## Abstract

Knowledge of connectivity and population structure is integral to the sustainable management of fished populations, yet such information is unavailable for many species over scales relevant to their exploitation. We examined broad-scale patterns and drivers of adult movement for a putatively mobile carangid (*Pseudocaranx georgianus*) on Australia’s southeast coast using an angler tag-recapture dataset. More than 6300 individuals were tagged and released across 1007 km of coastline, with anglers recapturing 157 (2.48%) individuals during a 14-year period. Median distance moved was 5 km and a substantial proportion of individuals (19%) were recaptured at their release location. Recapture latitude was also strongly predicted by release latitude (r^2^ = 0.87). However, a broad range of movements were observed (0–508 km), with 6% of individuals moving further than 100 km. Most individuals recaptured in areas now designated as Marine Protected Areas (MPAs) were originally released in the same area (79.2%). Larger body size, longer periods at liberty, and releases during Spring all positively influenced distance moved. Results support restricted movement over an intermediate scale, punctuated by occasional large movements. Our findings suggest adult movement of *P*. *georgianus* in southeastern Australia primarily occurs over smaller distances than the current spatial scale of management.

## Introduction

Knowledge of connectivity and population structure is essential for the sustainable management of fished populations^1^. In addition to local adaptation and genetic divergence, the extent of connectivity among subpopulations can influence demographic rates (e.g. recruitment) and therefore affect population persistence^2,3^. The extent of movement in older life stages can also influence biological characteristics (e.g. growth) across environmental gradients^4^, which may affect relative productivity among subpopulations. Management of fished populations at spatial scales that do not reflect scales of connectivity and resulting population structure can lead to localised depletions and demographic shifts^5^, particularly where subpopulations are differentially exploited^6^.

Despite the importance for management, scales of connectivity remain unknown for most exploited marine fishes^1^. The dispersal processes that drive connectivity among subpopulations are complex, differing among species and life-stages. For species with a sedentary adult stage and a pelagic larval stage, like many reef fishes, scales of connectivity are primarily determined by the extent of larval dispersal, which may be 100s to 1000s of km^7^. Larval dispersal and resulting connectivity have been a particular focus of research over the last two decades^8,9^. However, many exploited species also have a highly-mobile adult stage, capable of moving 100s of km within a lifetime^10^. Such movements may further complicate patterns of connectivity established during the larval stage and remain poorly understood for most species^11,12^. Knowledge of the patterns and extent of post-settlement movement is essential for a holistic understanding of connectivity for mobile species. Such information is also critical for informing and evaluating spatial management approaches like Marine-Protected Areas (MPAs)^13^.

Adult movement has traditionally been investigated using mark-recapture techniques, which provide individualised estimates of movement based on the distance between release and recapture locations^14^. However, mark-recapture investigations can suffer from low recapture rates, owing to dilution of marked individuals within the broader population. The power of such studies can be substantially increased through the participation of fishers^15,16^. Commercial and recreational fishers catch more individuals of target species than independent researchers and often do so over much broader spatial and temporal scales than can be achieved within a typical research program. This sampling effort can be harnessed through cooperative tagging programs, where fishers tag and release their catch using tags supplied by researchers. Fishers then report recapture events, including the location, date and size of the individual. Although cooperative tagging programs involve numerous potential biases^17,18^, they can provide data on post-settlement movement over large spatial and temporal scales that may not otherwise be cost-effectively obtainable^15,19^.

Trevallies from the genus *Pseudocaranx* (Bleeker, 1863) are an important fishery resource throughout subtropical and warm temperate oceans^20,21^, yet recent species revisions and uncertain population structure have complicated management efforts. Revisions in Australasia split the complex previously identified as *P*. *dentex* into three separate species; *P*. *georgianus* in southern Australia and New Zealand, a new species *P*. *dinjerra* in northwestern Australia, and possible *P*. *dentex* in northeastern Australia and Lord Howe Island^22^. An additional species may also be present in Norfolk and Kermadec (Raoul) Islands. The revisions substantially narrowed the previous distribution of *P*. *dentex*, which is now almost completely restricted to the Atlantic. *P*. *georgianus* now comprises most of the fishery for trevallies in both Australia and New Zealand and forms a substantial component of both commercial and recreational catches in Australia^23^.

Fisheries for *P*. *georgianus* are largely coastal, with the major fishing methods being commercial trawl, trap, purse-seine and set net fisheries in Australia and New Zealand^24,25^, as well as recreational line fishing^25,26^. Within Australia, almost all landings of *P*. *georgianus* are reported from southeastern Australia, where the fishery has declined markedly since the 1980s^27^. Commercial fisheries for *P*. *georgianus* in Australia are an order of magnitude smaller than New Zealand, averaging ~330 t p.a. since 2006^27^, whereas in New Zealand average landings have been >3,000 t p.a. during the same period^25^. Recreational harvests are an order of magnitude smaller than commercial harvests in both countries, with estimates of ~27 t in southeastern Australia^26^ and ~190 t in New Zealand^25^.

It is unclear whether current scales of fisheries management for *P*. *georgianus* in Australasia are appropriate for sustainable exploitation. Management in both Australia and New Zealand currently occurs at a scale of 100–1000 km, reflecting the extent of jurisdictional boundaries in Australia and defined fishing zones in New Zealand^28^. In Australia, total allowable catch (TAC) for the primary Commonwealth fishery, Southern and Eastern Scalefish and Shark Fishery (SESSF), is determined under the assumption of a common stock in the region^29^. Major fisheries for *P*. *georgianus* within the state of New South Wales (NSW) are managed assuming a single stock across the jurisdiction (1000 km of coastline). However, evidence that this scale matches scales of connectivity and resulting population structure is limited. In support, *P*. *georgianus* is a fast-swimming benthopelagic species with a shelf-wide distribution, from shallow estuaries through to the offshore environment. The species also has a pelagic larval phase, with early stage larvae (pre-flexion and flexion stages) being more abundant over the inner-shelf, while later stage larvae are more commonly found on the outer shelf^30^; likely the consequence of local hydrodynamics^31^, spawning periodicity (austral summer)^32^ and ontogenetics. Late-stage larvae are more commonly found in deeper water along the Australian east coast^33,34^, suggesting offshore mixing and potential genetic homogeneity in the region. However, tagging experiments in northeastern New Zealand indicate limited movement during the adult stage, with most recaptures occurring within 55 km of the release location^35^. Therefore, demographic population structure may exist over finer scales than that of current management.

We investigated the movement patterns of *P*. *georgianus* along Australia’s southeast coast using a large-scale tag-recapture dataset. Data were obtained from a recreational angling program covering 9.5° of latitude and spanning 15 years. Specifically, we investigated the effect of latitude, direction of movement (north or south), season, body size, and time at liberty on the likelihood of movement and the distance moved. This study provides the first information on the movement of *P*. *georgianus* in the region, which is essential for evaluating the spatial scale of current management strategies, including MPAs.

## Methods

### Tagging program

*P*. *georgianus* were tagged and recaptured by recreational anglers through the cooperative New South Wales (NSW) Fisheries Gamefish Tagging Program. The program engages anglers through angling clubs, with >190 clubs involved across the Indo-west Pacific region^17^. The program commenced in 1973 and is ongoing. Anglers are provided with tagging kits that include single-barb spaghetti tags, tag information cards and tagging instructions. The instructions indicate the correct technique for tagging and include diagrams demonstrating the insertion of tags behind the dorsal pterygiophores. Techniques for optimal fish handling are also provided, including placement of individuals on a cool wet surface and return of individuals to the water as soon as possible. Tag cards record the capture date, location, species, body length (total, cm), fish condition and angler details. Anglers are requested to provide the same information when they recapture a tagged individual. Tag cards and recapture details are provided to and managed by NSW Department of Primary Industries - Fisheries, either directly or through club representatives. Anglers are provided with certificates and information on the time at liberty and movement of the individual they tagged or recaptured. The tagging program was approved by the NSW DPI Animal Care and Ethics Committee (Permit No. 95/7) and has been conducted in accordance with requirements.

### Tagging location and period

**Figure 1:**
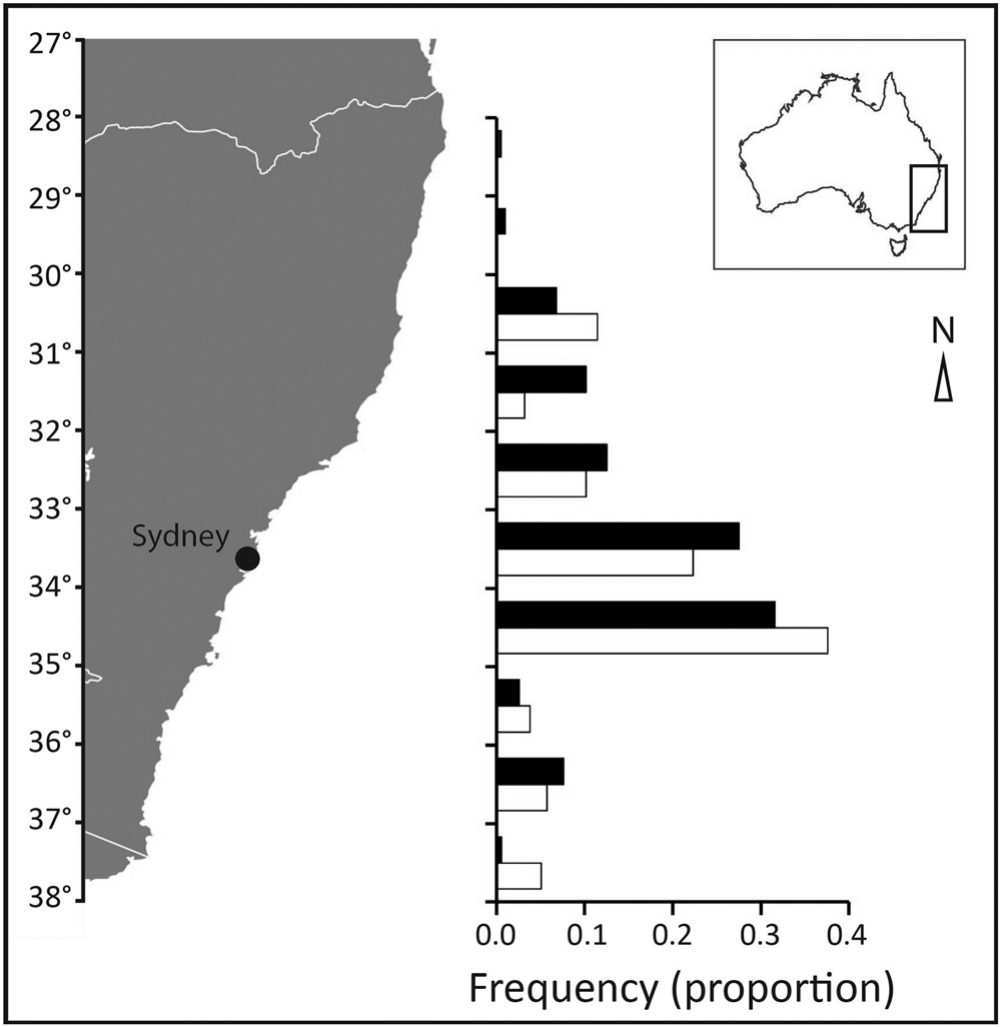
Map of Australia’s southeast coast indicating the proportion of releases (black bars) and recaptures (white bars) of *Pseudocaranx georgianus* across southern latitudes. White lines on the map delineate state borders.

Tag-recapture data was available for *P*. *georgianus* between 1984 and 1998. During this period, 6327 individuals were tagged and released along 1007 km of coastline, between latitudes 28 and 38°S (Fig. 1). Individuals were released on the open coast and inside coastal bays. Releases were unevenly distributed along the coast, with 59.1% of individuals released between 33 and 35°S, corresponding to the most densely populated region (Sydney, 33.865°S 151.209°E). Releases were also unevenly distributed through time, with 86% of individuals released between 1988 and 1990.

### Data processing

Data were checked for reporting errors prior to analysis. Location errors included implausible locations and mismatches between the names of locations and the coordinates provided. Implausible lengths were defined using published size information for the species and were considered to be <10 cm TL or >80 cm TL^36^. Subsequently, five recapture reports (3.1%) were excluded from further analyses.

The linear distance between release and recapture locations was used to estimate the distance moved (km) for each individual. Location coordinates were available to the nearest minute. This allowed resolution of distances within the study area to approximately 1.85 km on the north-south axis and approximately 1.49 km on the east-west axis. Therefore, individuals recaptured <930 m north-south and <750 m east-west from their release locations were recorded as moving 0 km.

### Data analysis

To examine general patterns of distribution along the NSW coast, the relationship between release latitude and recapture latitude was tested using simple linear regression. A two-stage hurdle model was then used to examine the potential drivers of movement. This approach was required because of the high proportion of ‘zero’ values for distance moved (see Results). The first stage used binary logistic regression to examine whether the odds of movement (‘yes’ or ‘no’) was influenced by time at liberty (in days, hereafter “Days”), body size at release (total length in cm, hereafter “Length”), latitude of release (in degrees, hereafter “Latitude”), or Austral season of release (Spring, Summer, Autumn, Winter, hereafter “Season”). An interaction term, Latitude*Season, was also included to account for potential differences in the effect of latitude among seasons. The second stage used negative binomial regression to examine whether the aforementioned factors, as well as the direction of movement (North or South, hereafter “Direction”), predicted the distance moved (km) for those individuals recaptured beyond their release location. This model included two additional interaction terms; Latitude*Direction and Direction*Season. These were included to account for the potential effects of latitude and season on the extent of directional movement. Data were explored prior to analyses using the protocol of Zuur *et al*.^37^.

An information-theoretic approach was used to identify models with the ‘best’ combination of explanatory variables, following the approach of Burnham and Anderson^38^. Model selection was based on Akaike’s Information Criterion corrected for small sample sizes (AIC_c_), with the lowest value indicating the best model. Models within two AIC_c_ values (∆AIC_c_ < 2) of the best model were considered to have strong support^38^. Model-averaging was then employed across models with ∆AIC_c_ < 2 to identify the most important explanatory variables, and to optimise coefficient estimates, using the ‘MuMIn’ package in R^39^. Data used in this study are available from the corresponding author on request.

## Results

Anglers recaptured 157 individuals, representing 2.48% of those initially released. Recaptures occurred during a 14-year period (1985–1998), with Days ranging from 1 to 2409 (6.6 years) and Length ranging from 16 to 70 cm TL.

The pattern of recaptures along the coast was similar to the pattern of releases, with the greatest number of individuals recaptured between 33 and 35 °S, corresponding to the most densely populated region (greater Sydney, Fig. 1). However, recaptures occurred across a narrower geographic range (754 km) than releases (1007 km), with no recaptures recorded from the two northern-most latitudes (Fig. 1).

**Figure 2:**
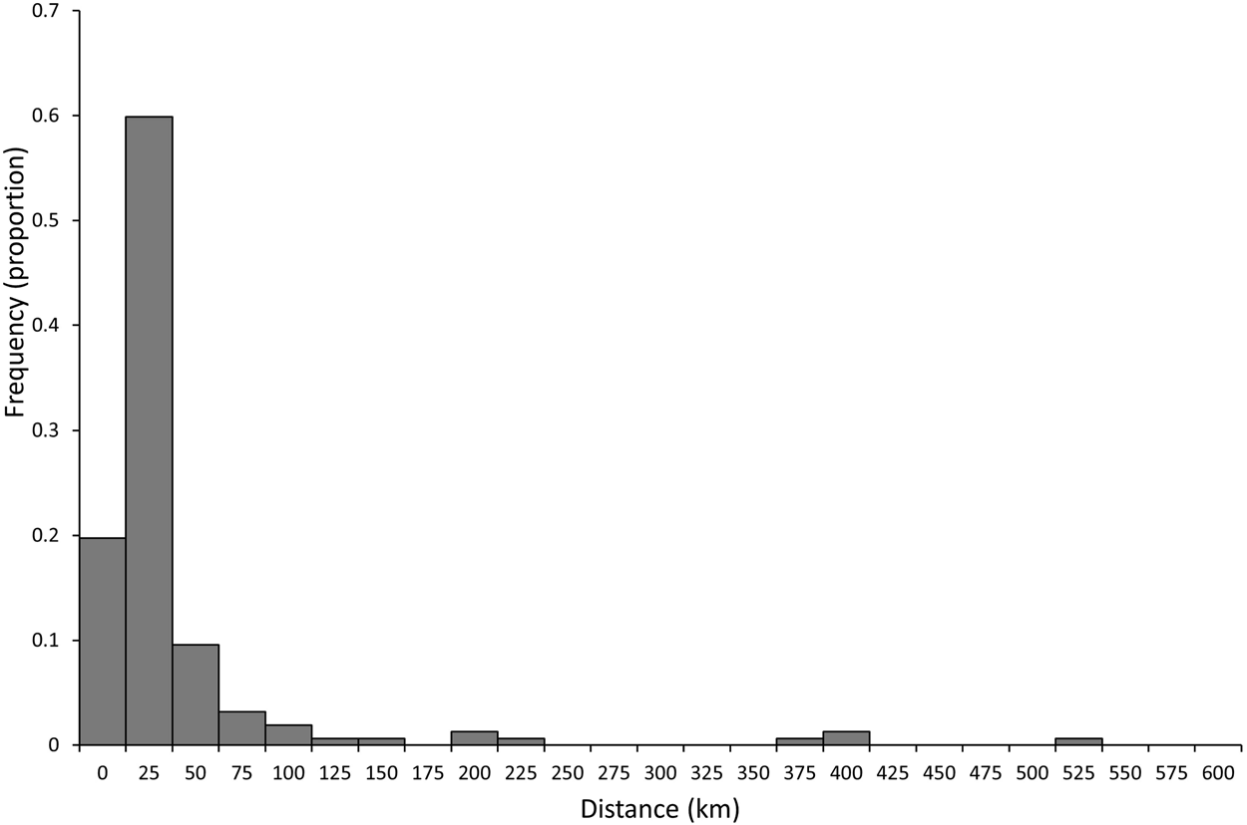
Distribution of distances moved (km) for *Pseudocaranx georgianus* across latitudes.

**Figure 3:**
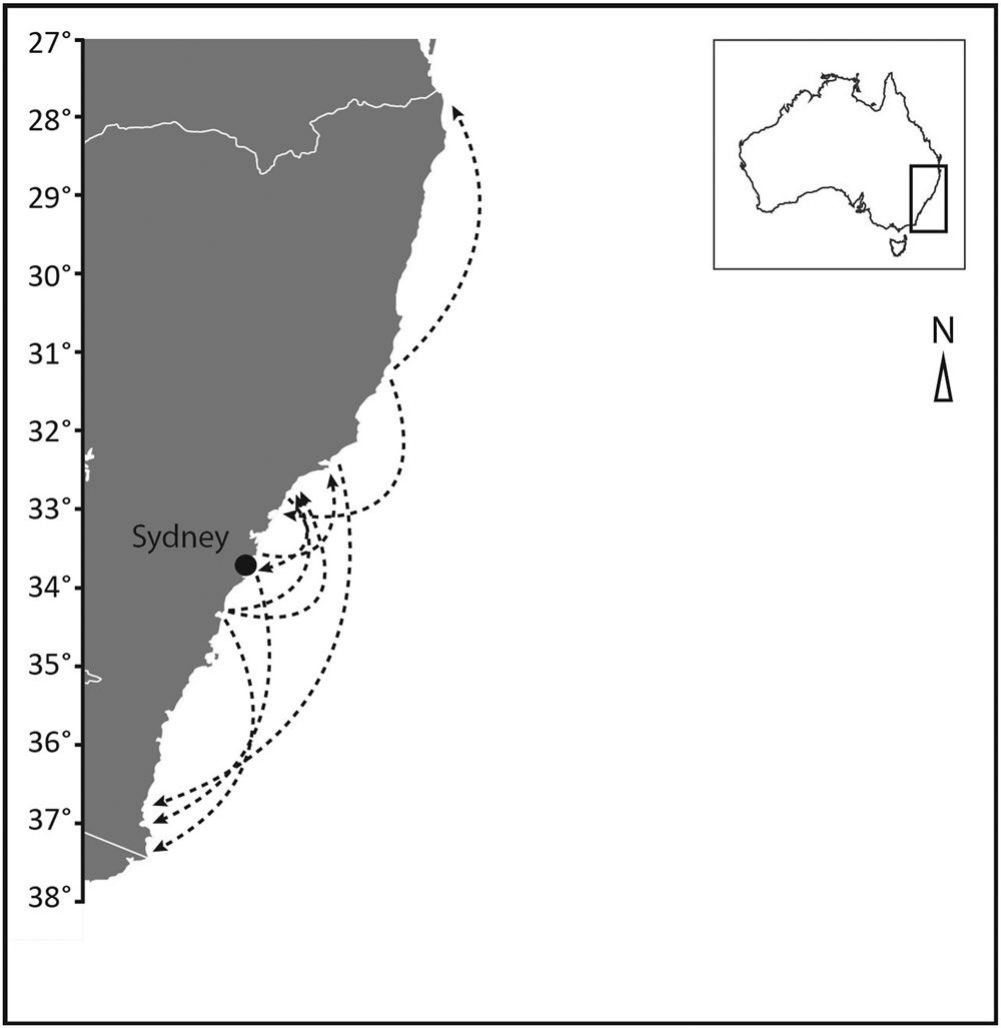
Largest movements of *Pseudocaranx georgianus* recorded along the NSW coast. These movements were undertaken by nine individuals (6%) and ranged between 119 and 508 km. White lines on the map delineate state borders.

Median distance between release and recapture locations was 5 km, which increased to 6 km when individuals at liberty <30 days were excluded. 61.1% of individuals were recaptured within 10 km of release, 76.4% within 20 km, and 88.5% within 50 km (Fig. 2). Recapture latitude was strongly predicted by release latitude (simple linear regression, r^2^ = 0.87, df = 156, p < 0.001), and this relationship changed little when individuals at liberty for <30 days were excluded (r^2^ = 0.85, df = 134, p < 0.001). A large proportion of individuals (19.7%, 31 individuals) were recaptured at their release location (<1.85 km), with time at liberty in this group ranging from 1 to 427 days. This proportion reduced to 18% when individuals at liberty for <14 days were excluded, then to 14% when individuals at liberty <30 days were excluded. Some individuals moved large distances (Fig. 2), with 9 individuals (6%) recaptured >100 km from their release point (Fig. 3). The largest movement of 508 km was made over 17 days (average speed: 1.25 km.h^−1^) by an individual of 63 cm TL.

Twenty-four individuals (15.3%) were recaptured within areas now designated as Marine-Protected Areas (MPAs). Of these, 19 individuals (79.2%) had been released within the same area. Time at liberty for these individuals ranged from 1–323 days and distances moved ranged from 0–17 km. A further three individuals (1.9%) were released in areas now designated MPAs, but recaptured outside of MPA boundaries. The MPAs are Solitary Island Marine Park (710 km^2^), Port Stephens Great Lakes Marine Park (980 km^2^), and Jervis Bay Marine Park (215 km^2^), which were established in 1998, 2005 and 1998, respectively.

Model selection procedures identified four candidate models to explain the odds of movement (∆AICc < 2, Table 1a). These models included three explanatory variables, with Days being the best predictor (relative importance: 1.0), followed by Latitude (relative importance: 0.43), then Length (relative importance: 0.32). Model-averaged coefficients indicated a positive effect of Days on the odds of movement (Table 2a), with each additional day at liberty increasing the odds of movement by a factor of 1.004. The effects of Latitude and Length were not significant at the 0.05 level (95% confidence intervals spanning 1, Table 2a). Overall, selected models explained little additional deviance (7.2–8.7%) in the odds of movement relative to the null model that included only the intercept (Table 1a).

**Table 1:** The best model candidates for (a) the odds of movement (‘yes’ or ‘no’) and (b) the distance moved (km) selected using AICc.

Model	df	AICc	∆AICc	Weight	Null deviance	Deviance explained (%)
**(a) Odds of movement**						
~Days	2	147.6	0.00	0.41	154.7	7.2
~Latitude + Days	3	148.4	0.77	0.28	154.7	8.0
~Days + Length	3	149.4	1.80	0.16	154.7	7.4
~Latitude + Days + Length	4	149.6	1.91	0.16	154.7	8.7
**(b) Distance moved**						
~Days + Length + Season	7	982.92	0	0.48	181.6	28.2
~Days + Direction + Length + Season	8	983.86	0.94	0.30	183.3	29.0
~Days + Latitude + Length + Season	8	984.49	1.58	0.22	182.5	28.6

Binary logistic models were used to model the odds of movement, while negative binomial models were used to model the distance moved. df indicates the number of model parameters.

**Table 2:** Model-averaged coefficients for models of (a) the odds of movement and (b) the distance moved (km).

Model term	Estimate	Confidence interval	
		2.5%	97.5%
**(a) Odds of movement**			
(Intercept)	0.212	0.000	400.439
Days	1.004	1.001	1.008
Latitude	1.067	0.860	1.324
Length	1.005	0.977	1.034
**(b) Distance moved**			
(Intercept)	3.312	0.085	129.454
Days	1.001	1.000	1.002
Length	1.035	1.013	1.056
SeasonSpring	2.022	1.055	3.874
SeasonSummer	1.458	0.683	3.112
SeasonWinter	0.510	0.270	0.964
DirectionS	1.087	0.763	1.547
Latitude	1.017	0.919	1.124

Estimates are back-transformed values indicating (a) odds ratios of movement, and (b) multiplicative effects on the distance moved. If confidence intervals include 1, there is no significant effect of the model term on the dependent variable at the 0.05 level.

For distance moved, model selection procedures identified three candidate models (∆AICc < 2, Table 1b). These models included five explanatory variables, of which Days, Length, and Season were the equal best predictors (relative importance: 1.0), followed by Direction (relative importance: 0.30) and then Latitude (relative importance: 0.22). Model-averaged coefficients indicated a positive effect of Days and Length on the distance moved (Table 2b), with each additional day at liberty increasing the distance moved by a factor of 1.001 and each additional cm of body length increasing the distance moved by a factor of 1.035. Releases in spring increased the distance moved by a factor of 2.022 relative to autumn, while releases in winter decreased the distance moved by a factor of 0.510 relative to autumn (Table 2b). The effects of Direction, Latitude and summer releases (relative to autumn) were not significant at the 0.05 level (95% confidence intervals spanning 1, Table 2b). Selected models explained 28.2–29.0% additional deviance in the distance moved relative to the null model that included only the intercept (Table 1b).

## Discussion

*Pseudocaranx georgianus* is a commercially- and recreationally-important species in southeastern Australia^21^, yet management has operated without detailed knowledge of connectivity and population structure. Despite fast swimming ability and offshore benthopelagic behaviour, our results indicate that movement of adult *P*. *georgianus* is likely restricted over the scale of fisheries management in the region (~1000 km), with a median distance between tag and recapture locations under 10 km and a high proportion of recaptures occurring at release locations. The findings suggest the existing management scale should be re-examined, particularly given the declines in landings observed in southeastern Australia since the 1980s^27^. Potential variation in catch histories and population responses among areas within southeastern Australia should be investigated to ensure that stock assessments and management approaches currently enacted at a regional scale are consistent with patterns occurring at smaller scales.

Results from this study indicate an intermediate scale of movement for *P*. *georgianus* in southeastern Australia, which is consistent with a tag-recapture study in northeastern New Zealand, but contrasts with acoustic tracking conducted in Australia. In New Zealand, 57% of tagged *P*. *georgianus* individuals were recaptured within 18.5 km of release and 88% were recaptured within 55 km of release^35^, similar to the distances observed in this study. However, acoustic tracking of *P*. *georgianus* in Western Australia indicated small home ranges (≤1.3 km^2^), with all but one individual remaining within the receiver array (4 km^2^) for >105 days and up to 356 days^40^. Acoustic tracking of six *P*. *georgianus* individuals within a Sydney estuary also identified small core areas (≤4 km) for most individuals^41^. The apparent disparity in the scales of movement between these studies may reflect differences in movement behaviour among locations or habitats, with smaller movements occurring in southwestern Australia and a southeastern Australian estuary, or it may reflect difference in the biases between tag-recapture and acoustic tracking techniques. The two tag-recapture studies extended over multiple years, allowing greater time for larger movements to occur and be detected. The acoustic tracking studies were restricted by the locations of receivers and to individuals inhabiting small inshore patch reefs or estuaries^40,41^, which may not reflect the movement behaviour of individuals further offshore. The scale of movement of *P*. *georgianus* is comparable to congener *P*. *dentex*, but may be larger than other members of the subfamily Carangini investigated to date. Acoustic tracking of *P*. *dentex* in the Azores Islands identified relatively large short-term movements compared to other reef species investigated to date (up to 29 km)^42^. More restricted movement has been identified for *Caranx melampygus*, with 75.5% of tagged individuals recaptured within 0.5 km of their release locations in Oahu, Hawaii^43^. Acoustic tracking of *C*. *ignobilis* in northeastern Australia and Hawaii also revealed high fidelity to home reefs and few inter-reef movements^44,45^. Comparisons among species are made cautiously here, given the different scales of investigation among studies. Larger movements may be identified for other trevally species if the broader temporal and spatial scales employed in this study are replicated in future investigations.

Movement distances observed for *P*. *georgianus* in this study indicate the potential for demographic structuring of the southeastern Australian population. Only 6% of adults were recaptured >100 km from their release point, indicating locations separated by greater distances experience limited exchange. Exchange of adults is therefore likely restricted between at least some subgroups of the population, given the extended geographic range of *P*. *georgianus* along the coast (~1000 km, Fig. 1). Similar to other tag-recapture studies, the total distance moved by individuals in this study has likely been underestimated to some extent, because individuals are expected to have moved further than the straight-line distance between release and recapture locations^10,46^. However, data presented here still inform the scale of adult exchange over longer time periods, because individuals that moved larger distances but were recaptured closer to their release location have not permanently left this area. *P*. *georgianus* in southeastern Australian potentially exhibit site fidelity and homing behaviour observed in conspecifics from southwestern Australia^40^, and other trevally species (e.g. *C*. *melampygus*, *C*. *ignobilis*)^43,44^, which could be tested using finer-scale acoustic tracking in the region. Such investigations would provide a useful complement to the current tag-recapture dataset.

Structuring of the adult population may lead to variation in demography along the coast, driven by spatially-variable environmental conditions, differential fishing pressure, or a combination of both^47^. For example, the large gradient in water temperature from north to south may reduce growth rates, and therefore productivity, in southern (poleward) areas relative to northern areas^48^, but see^49^. Areas of higher fishing pressure may also experience truncated length and age structures through loss of larger, older individuals that are subsequently not replenished^50^. Length structures from commercial trawl catches in southeastern Australia during 1997–1999 indicated a pattern of increasing body size with latitude, yet the cause of this pattern is unknown^21^. Further, there is evidence of a temporal change in length structure of *P*. *georgianus* in this region, with substantial declines in the length of landed catches from the late 1980s through to the late 1990s^27^. Investigation of the potential demographic consequences of limited adult exchange is required to predict the effects of future fishing pressure and environmental change on population demography.

In contrast to demographic structure, restricted adult movement is unlikely to influence the genetic structure of *P*. *georgianus* in southeastern Australia. *P*. *georgianus* have pelagic eggs and larvae that are spawned on the open coast^32^. These stages are likely dispersed over far greater distances (100s-1000s km) than adult movements, particularly given the strong southward flow of the East Australian Current (EAC) during the summer spawning season^51^. Substantial genetic structure is therefore unlikely along the coast, but if present, controlled by patterns of larval connectivity, rather than adult movement.

A strong relationship was found between Season and movement for *P*. *georgianus* in the current study, with releases in spring having a strong positive effect on the distance moved. Increased movement in spring may be related to migration prior to the summer spawning season in southeastern Australia^21,30,32^. A southward spawning migration has been suggested for *P*. *georgianus* in the region based on reports from the purse seine fishery^21^. However, this has not been empirically demonstrated, and the current study did not find a significant effect of direction on distance moved, nor was the interaction between direction and season an important model term. An alternative explanation for increased movement during spring relates to increases in feeding activity driven by an increase in water temperature later in the season, upwelling and coastal productivity in the region^52,53^.

The large variation in movement distances among individuals suggests multiple behavioural types may exist for *P*. *georgianus* within southeastern Australia. The movement of one individual over 500 km within 17 days indicates the potential mobility of the species, yet numerous individuals were recaptured at their release location 100s of days later. Some of this variation can be accounted for by body size, with predicted movement distance being 57 km greater at the largest size encountered in the current study relative to the smallest size, based on the upper limit of the coefficient estimate for Length and holding other model terms at zero (Table 2). However, the remaining variation may partially be explained by behavioural differences, with some individuals being relatively sedentary, or exhibiting strong homing behaviour, while others have less site fidelity and undertake extensive migrations. Such differences in movement behaviour within populations were first recognised in species that undertake extensive diadromous migrations (e.g. salmonids)^54^, but are increasingly being reported for marine species^55^. Knowledge of the prevalence of different movement behaviours within populations is critical for understanding the effect of spatial management approaches, particularly the level of protection offered and the extent of export from MPAs^11,13^. Our tag-recapture results from areas now designated as MPAs along Australia’s southeast coast suggest these areas afford the less-mobile contingent of the population some degree of protection from fishing, but dedicated investigations in these areas involving greater replication would be required to demonstrate this with a high level of confidence. If a substantial proportion of the *P*. *georgianus* population remain within MPAs and are not available to fisheries in the region, it is possible that catch quotas assuming their availability have been set too high in recent years^24^.

The angler-based tag-recapture dataset used in this study allowed investigation of movement at temporal and spatial scales rarely achieved in fishery-independent investigations. The large spatial scale is particularly useful for investigating species capable of long-distance movements like *P*. *georgianus*, because there is a greater likelihood that long-distance movements will be captured. However, such datasets often have specific biases and limitations that reduce their utility relative to other approaches^17^. One potential bias involves uneven (and unknown) angler effort that distorts patterns of release and recapture^56^. In this study, it was clear that locations closer to Sydney, a major urban centre, were more heavily targeted than regional locations (Fig. 1). This could have resulted in shorter distance estimates, because individuals released in the Sydney region were more likely to be recaptured there than at locations further away. While the full effect of this potential bias cannot be resolved here, latitude was not found to be a significant predictor of distance moved, suggesting movement estimates near Sydney were not substantially smaller than elsewhere. Angler surveys are required to understand spatial and temporal patterns in effort for *P*. *georgianus* in the region and these estimates may help inform possible bias.

Angler tag-recapture studies may also result in higher post-release mortality and tag shedding than fishery-independent investigations, due to improper handling and tagging procedures. Although neither of these issues could be addressed in the current study, *P*. *georgianus* are not susceptible to barotrauma^57^, so likely survived releases that were completed within a timely manner. Also, while poor handling and tagging procedures may reduce the recapture rate, these issues are not expected to result in biased estimates of movement distance.

Our findings support an increasing body of evidence that fishes with strong swimming ability can exhibit limited adult movement, leading to demographic population structure. In southeastern Australia, the paradigm that species inhabiting offshore waters represent single unit stocks for management purposes, based on the strong southward flow of the EAC and observations of individuals moving substantial distances, is being increasingly challenged as more detailed stock structure studies are completed^58,59^. Knowledge of such patterns is particularly important where multiple jurisdictions access a common resource and the scales of multiple management regimes may not adequately reflect population structure. The current study demonstrates how tag-recapture datasets can contribute to our understanding of the demography of fast-swimming species with broad distributions, complementing more detailed approaches such as acoustic telemetry. Such tag-recapture data are unlikely to be obtained without the support and active management of angler-assisted tagging programs.
